# Yoga for Children and Young People’s Mental Health and Well-Being: Research Review and Reflections on the Mental Health Potentials of Yoga

**DOI:** 10.3389/fpsyt.2014.00035

**Published:** 2014-04-02

**Authors:** Ingunn Hagen, Usha S. Nayar

**Affiliations:** ^1^Department of Psychology, Norwegian University of Science and Technology, NTNU, Trondheim, Norway; ^2^New School University, New York, USA

**Keywords:** children, young people, mental health, well-being, yoga

## Abstract

This article discusses yoga as a potential tool for children to deal with stress and regulate themselves. Yoga provides training of mind and body to bring emotional balance. We argue that children and young people need such tools to listen inward to their bodies, feelings, and ideas. Yoga may assist them in developing in sound ways, to strengthen themselves, and be contributing social beings. First, we address how children and young people in today’s world face numerous expectations and constant stimulation through the Internet and other media and communication technologies. One reason why children experience stress and mental health challenges is that globalization exposes the youth all over the world to various new demands, standards, and options. There is also increased pressure to succeed in school, partly due to increased competition but also a diverse range of options available for young people in contemporary times than in the past. Our argument also partially rests on the fact that modern society offers plenty of distractions and unwelcome attractions, especially linked to new media technologies. The dominant presence of multimedia devices and the time spent on them by children are clear indicators of the shift in lifestyles and priorities of our new generation. While these media technologies are valuable resources in children and young people’s lives for communication, learning, and entertainment, they also result in constant competition for youngster’s attention. A main concept in our article is that yoga may help children and young people cope with stress and thus, contribute positively to balance in life, well-being, and mental health. We present research literature suggesting that yoga improves children’s physical and mental well-being. Similarly, yoga in schools helps students improve resilience, mood, and self-regulation skills pertaining to emotions and stress.

## Introduction

Globalization exposes children and young people all over the world to various new standards and options. Now children not only have new resources in their lives, but are also expected to perform well. Different institutions in children and adolescents’ lives, such as family, school, and the media, constantly provide stimulation as well as expectations. This exposure to new expectations and demands has the potential to create stress in young people’s lives, especially related to evaluation of their performances.

Recent research shows that the most stressed-out generation is the current young adults (1). For example, the 2012 online survey “Stress in America” reported an average stress level of 5.4 out of 10 among 2,020 respondents in the US who were 18–33 years old. The researchers considered a stress level of three to six to be healthy (2). The survey has found that millennials (18–33 years of age) are more stressed than any other current living generation. Respondents in the millennial generation were also less likely to give their healthcare an A grade. Almost half of them acknowledged not

It is common knowledge that stress can have serious health consequences. If unaddressed consistently, a high stress level could become a chronic condition, which could result in a range of health problems, including anxiety, insomnia, muscle pain, high blood pressure, and a weakened immune system. Research indicates that stress can even contribute to the development of major illnesses such as heart disease, depression, and obesity or exacerbate existing health issues (1). When such young adults are responsible for child care, they may tend to transmit their tensions to their children; thus, the situation becomes doubly alarming and worrisome for their families. Children learn to internalize the stresses. Their self-imposed expectations to meet the standards set by their caregivers, schools, and society may cause them anxiety. Moreover, internalization of self-expectation may become non-malleable for young people.

We have observed that children are quite good at hiding their distress and emotional stress from their parents, since they do not want their parents to worry on their account. They desire to please their parents by their “appropriate” and “socially right” behaviors. Children dislike upsetting their parents and being the reason for adding to existing parental stress. According to a constructivist approach, children actively participate in their own development process. Moreover, children and young people interact with everyday life situations with world views that could be different from those of adults. In line with this theory, we believe that children function as an agency for their own well-being and have the evolving capacity to be partners of wellness with their families, friends, and society. However, children depend on the environment set by society to facilitate their potential for development.

This article discusses yoga as a potential tool for the youth to deal with stress and to regulate themselves. Yoga provides training of mind and body to bring emotional balance. It is claimed that yoga leads to alignment and harmony. A recent thesis suggests that yoga is a tool to listen to your heart (3). We argue that children and young people need such aid to listen inward, to their bodies, feelings, and ideas. Thus, yoga may contribute to healthy development and good mental health; health promotion for children needs to include improvement of their attention, self-esteem, empowerment, and self-regulation. We believe that children and adolescents need to develop based on their unique personalities, and to interpret and achieve the balance between their own strengths and societal expectations. Yoga may assist them in developing in sound ways, to strengthen themselves, and be contributing social beings.

## Children, Young People, and Mental Health

There are also examples of today’s youth who demonstrate more serious attitudes than those of earlier generations. For instance, in Norway, as in some European countries, youngsters use less drugs than did previous generations, perform better academically, and are more active participants in society and in elections (4). Moreover, youth behave more decently and less criminally, and are more hard-working in school. Children and young people also share more values with their parents than did their pre-decessors, and while young people “hang out” online, many from the parent generation do the same. This similarity may be one of the reasons for the decrease in drug use and breaking of norms. However, this amicable behavior has its price; recent research also indicates that young people are more worried than their counterparts in the past (*op. cit*.). Furthermore, a greater number of youth are diagnosed with conditions such as attention deficit hyperactivity disorder (ADHD). Increased pressure is also exerted on them to succeed in school now than in previous times. Such increased emphasis on education and self-discipline can be challenging. Thus, there are more psychological problems among young people; many worry excessively, have sleep problems, and experience hopelessness and stress.

In fact, mental health problems are common among children and young people in the West, as well as in other parts of the world. The 2012 European Union (EU) Youth Report suggests that almost 10–20% of young people in Europe suffer from mental illnesses, while one out of five struggle with emotional or behavioral problems^1^. Other sources confirm that while the majority of adolescents worldwide are healthy, 20% experience mental health issues (5). Similarly, a recent report estimates that 15–20% of Norwegian children aged 3–18 years have reduced functioning abilities due to mental problems such as anxiety, depression, and behavioral disorders (6). Generally, psychological challenges intensify around puberty; due to psychological and biological changes. When growing up, children face pressures from family, school, and other social contexts to perform satisfactorily and adjust to the rapidly changing pace of development in society.

The current scenario is challenging for both teachers and parents, as well as for children, to foster a positive mental health status. The transition from early childhood to youth and adulthood can be demanding in itself. In the midst of dealing with physical changes, children also have to develop their own identity, increase their autonomy from their parents, and handle changing peer relations. The pressure on young people also varies, encompassing academic, commercial/marketing, and relational issues, as well as succeeding in school, being popular, having a fit or slim body, wearing the right brands of clothes, and owning the latest technological gadgets, etc. (7). This set of expectations creates stress, which impacts children and young people’s mental health and well-being, as well as hampers their school performance [see Ref. (8)].

Children also suffer from bullying (at school and cyberbullying), behavioral issues, problems with attention and self-regulation [such as ADHD and attention deficit disorder (ADD)], sleep disorders, obesity, computer dependency, drug abuse, and lack of school motivation, even leading to dropouts. Recent dropout rates in high schools are close to 30%, despite several years of political priority and designated measures in both the EU and the United States (US). Furthermore, schools are faced with the challenge that students (especially boys) are more attracted to the Internet, social media, and gaming than the school curriculum [cf. Ref. (9)].

## Children and Young People’s Media Use and Health Challenges

Modern society also offers innumerable distractions and undesired attractions, especially linked to modern media and communication technologies, on which we have become dependent. The massive presence of media and the time spent on media technologies by children are clear indicators of the shift in lifestyles and priorities of our new generation^2^. In the US, children spend over seven and a half hours daily using media devices (10), an alarmingly large average, yet rather matter of fact in 2013. Children worldwide are spending more and more time in front of television sets or computer screens and on cell phones, making media a central part of their lives. Young people today are expected to be and are often constantly online. Advertising, communications, as well as education present a brand new social networking image to make media accessible to children^3^.

Although media is a knowledge resource for children and adolescents’ mental health, its intense use leads to questions concerning young people’s capacity and interest to bring balance between physical and mental activities. A Kaiser Family Foundation study examined media use among very young children (0–6 years) and concluded that even these children spend as much time with electronic gadgets as they do playing outside (11). This study and many others draw links between media use and increase of health issues such as obesity and other physical and mental problems. Healthcare professionals use terms such as media addiction, identifying media as a factor of mental illness, dependency, obsessive–compulsive behaviors, concentration problems, and other attention disorders. Besides these physical and mental risks, safety concerns are being raised in media-heavy communities; issues such as cyberbullying, young children being exposed to violence, and sexually explicit material, as well as extreme or inappropriate behaviors, are being highlighted. The world at large, including the deviances of society, is much closer and easily accessible with media tools and technologies.

We understand the media generation as the youth who live in a hypermedia environment. In many countries, including India, unequal access to media is an important concern, especially, since information and communication technologies (ICT) are regarded as major knowledge resources for the future (12). Children and young people’s media use needs contextualization if this practice is to be understood properly [cf. Ref. (13, 14)]. First of all, children’s everyday lives, which include their home situation, school, and leisure activities, provide a context. Cultures and norms are also contexts to consider when understanding the role of new media and ICT in children’s lives. Media use is related to young people’s social context, such as family, community, and friends or peer groups. The media landscape, including traditional media, is also the context for how new ICTs are appropriated. Children’s use and reception will mediate the potential impact of media exposure. The consequences of media use can be extensive, and may affect how children spend their time, socialize, and even view the world. Thus, young people’s media use can be a factor in how they experience themselves and their lives.

## Yoga in Children’s Lives

The ancient practice of yoga may help children and young people cope with stress and thus contribute positively to mental health. In a recent book on yoga education in India, the author claims that “in a nutshell, yoga is a powerful medium for developing the personality of children and making them capable of facing the present-day challenges and problems” [(15), p. 3]. In her review article, “Effect of Yoga on Mental Health in Children,” one of the world’s most prominent yoga researchers, Shirley Telles, concludes that yoga improves children’s physical and mental well-being (16). Similarly, the Harvard professor Sat Bir Khalsa (17) finds that yoga in schools helps students improve resilience, mood, and self-regulation skills pertaining to emotions and stress. Thus, yoga is an important life skill tool for children and young people to cope with stress and self-regulation in a life-long perspective.

As researchers and responsible citizens concerned with children and young people’s healthy development, what can we do to provide a happy environment and opportunities for them to develop their maximum potentials? With this profound question confronting us, we would like to provide the arguments for choosing yoga. Short-term solutions oftentimes involve pharmaceutical treatments for children with mental health problems, which could leave them to face the immediate and long-term negative effects of medication (18). Thus, we must seek other solutions comprising empowerment to give children and young people the tools to develop self-reflection, self-protection, self-regulation, and holistic self-development.

The increased global interest in yoga in recent decades is primarily due to the expectancy that yoga can calm the mind and increase overall health and well-being. Children’s mental health and well-being include developing healthy relationships with peers and teachers, and being able to self-regulate emotionally, mentally, and behaviorally [see Ref. (16)]. Yoga is an ancient Indian practice, which has been spread all over the world, and is even being revitalized in India itself. Yoga consists of certain postures (*asanas*), regulated breathing techniques (*pranayamas*), hand poses (*mudras*), and meditation. There is experiential knowledge on which poses are appropriate for different bodily functions. Yoga’s positive impact on the physical and mental health of individuals and their well-being has been an established truth in the ancient as well as contemporary yoga literature [e.g., Ref. (19)]. The recent scientific research on yoga provides empirical evidence for some of these claims, and specifies that certain yoga practices are beneficial for the mental and physical health of children and young people [see Ref. (20, 21)].

Yoga can be an appropriate scientific intervention in childhood and youth as a stress alleviator, especially in the school setting. The mentioned study conducted by Khalsa (17) on high school students does provide evidence of yoga’s positive influence on them for emotional balance and stress alleviation. Yoga is also expected to help younger children and youth increase self-regulation and thus, facilitate their well-being, positive social interactions, and school performance. Other academic research suggests that yoga has significant health potentials and is especially beneficial for coping with stress (22). A meta-analysis of articles suggests that “a growing body of evidence supports the belief that yoga benefits physical and mental health via down-regulation of the hypothalamic–pituitary–adrenal (HPA) axis and the sympathetic nervous system” [(23), p. 3]. The use of yoga among children may assist their development, increase their well-being, reduce everyday stress, facilitate weight management, and mitigate emotional and behavioral problems, aside from being a supplement to improve focus and attention.

The evidence of yoga practice among children indicates improved benefits in concentration, stress alleviation, self-awareness, consciousness, self-regulation, behavioral and emotional maturity, and self-confidence in everyday life. There are also some proofs where yoga has worked as an adjunct to medical treatment of mental illness with positive effects. Yoga as a stand-alone therapy requires further research, although there are quite affirmative indications. In their review article, Büssing et al. (20) claim:

*Yoga may well be effective as a supportive adjunct to mitigate some medical conditions, but not yet a proven stand-alone, curative treatment. Larger-scale and more rigorous research with higher methodological quality and adequate control interventions is highly encouraged because yoga may have potential to be implemented as a beneficial supportive/adjunct treatment that is relatively cost-effective, may be practiced at least in part as self-care behavioral treatment, provides a life-long behavioral skill, enhances self-efficacy and self-confidence and is often associated with additional positive side effects* (2012: 1).

Despite such assertions, we need more scientific research enriched with demonstrative practice among children.

## Potential Benefits of Practicing Yoga for Children and Adolescents

“The beauty of yoga is that its benefits are available to students of every school-age group,” according to Henningsen (24). She discusses how yoga can be a comprehensive approach to stress, something which is needed in the often tension-filled lives of children today [see also Ref. (25)]. Yoga can help foster motivation, cultivate internal locus of control, improve sleep, and generally encourage healthy and balanced living. Yoga may also aid in shifting self-awareness inward to children’s own cues and emotions, and thus, counteract negative social and cultural influences, including the current media pressure to be always online and available. As yoga often results in improved focus and concentration, regular practice is frequently accompanied by better academic performance (26). Yoga has also been shown to help children with attention problems (27), as well as to support executive function development (28). A number of studies have also suggested that yoga can assist children with special needs.

Yoga has been found to have physiological benefits for children through rehabilitation processes (29). Clinical studies also indicate that yoga improves academic performance and emotional balance [see Ref. (30)]. The mental benefits of yoga relate to calming the heart rate, which signals the brain to activate the parasympathetic nervous system. Similarly, yoga can guide relaxation because it reduces sympathetic activity [e.g., see Ref. (31)]. The sympathetic nervous system (fight or flight) is often engaged when children, similar to adults, are exposed to sensory overload. However, when the parasympathetic nervous system is activated, it increases our ability to focus and learn. Yoga is also said to reduce anxiety in young people as well as in seniors (32). Thus, yoga may assist healthy development and life-long learning.

As already mentioned, yoga has been recommended to promote mental health (16, 21) and to increase resilience and self-regulation (17, 33). It has been researched in areas such as life-span development (34), human attachment (35), elderly subjects (36), consciousness (37), as well as in the medical and psychological fields (38–43). We think that what is still needed is a set of recommendations on how to introduce yoga into children and young people’s lives, based on a cultural perspective on child development and childhood sociology. Yoga is often promoted as a universal good discipline, with philosophy and practice to achieve balance and human development. Still, the pedagogy of yoga needs to be context specific and adjusted to the specific audiences of practitioners. Moreover, we believe that yoga teaching needs to be serious and multi-disciplinary, yet based on children’s needs and everyday lives, with a playful and fun approach.

**Figure 1:**
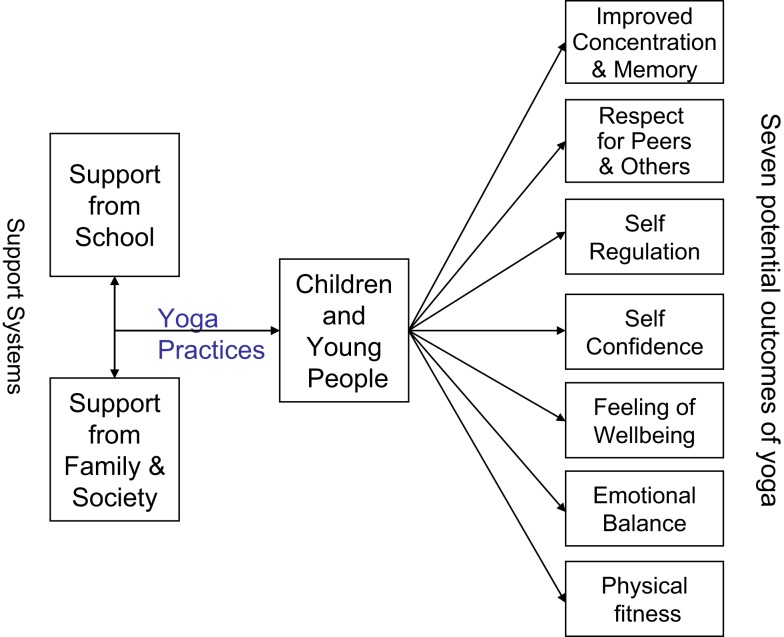
**Yoga for Young People’s Mental Health and Wellbeing**.

Figure 1 illustrates some potential outcomes of yoga practice for children.

## Discussion

This article claims that yoga can be a valuable tool for children. We believe that the following statement also applies for children and young people: “If you practice yoga every day with perseverance, you will be able to face the turmoil of life with steadiness and maturity” [(19), p. 127]. The business enterprise has recognized the value of yoga globally. Across urban areas in recent times, yoga training centers, practice centers, private agencies, and individuals for both profit and non-profit sectors have opened studios and organized sessions in various forms and approaches. Many people pay fees to use these facilities and practice yoga. However, schools, pre-schools, and the public sector of education are not keeping up with the trend, notwithstanding their prime responsibility of developing the full potentials of children and young people.

Today’s children require a creative, interactive syllabus, and participatory method in the teaching–learning process. This approach is applicable for learning yoga too. Thus, if we can communicate with children and young people effectively, they can adopt yoga as a powerful tool for themselves to minimize stress, as well as develop resilience to deal with it. We believe in the need to focus on research to understand the ways children and young people can enjoy learning yoga, sustain it in practice, and use it in daily life. They may use yoga in any kind of emotional and social stress situations. From a social perspective, we anticipate that yoga can also transform people to be socially sensitive; hence, it may increase the likelihood of children and young people engaging in civic activities and shaping a better society. As stated, practicing yoga has the potential to improve the mental health of children and young people. We have argued that children and young people are agents who possess the evolving capacity to develop the self and society, and yoga is a means to attain holistic well-being for both. We may bring this conceptual value framework of individual and society into a continuum of restoring social democratic systems for yoga and mental health of children and young people.

In this article, we have addressed some stressors to which children are exposed in their everyday lives in modern societies, including rising expectations and children’s extensive media usage. We have claimed that children and young people need yoga for stress management, self-regulation, and healthy development. As mentioned earlier, research on the effects of yoga on children and young people’s mental health and well-being is at an early stage. When introducing yoga to children, we think it is important to keep in mind what Shakta Khalsa – a pioneer in teaching yoga to children suggests: children’s yoga is not a simplified version of yoga for adults, it is unique practice. Khalsa also emphasizes that it is important to meet children where they are, and that they experience yoga as fun. The basic motivation of teachers should be that yoga strengthen children’s self-esteem and focus through their consciousness of themselves from within [see Ref. (44)].

### Research gaps

Only over the last 10 years have some researchers been engaged in the study of yoga’s beneficial effects on the youth’s health and well-being, and this area needs further development. Yoga research is a promising field but is currently dominated by medical research [e.g., Ref. (45)]. This research is very valuable, and generally supportive of the potential positive impact of regular yoga practice in children and adults lives. However, there is also a need to reveal the nature and type of impact of yoga practice on children, from a psychological perspective. Additionally, there are gaps in the research pertaining to the relationship between various yoga techniques/practices and mental health benefits. There is also a lack of empirical evidence evaluating the correlation between specific yoga practices and developmental milestones among young people.

### Recommendations

We think that yoga could provide tools for children and young people to remain centered or regain focus, so they may cope with the stress and challenges they experience in their everyday lives. Such tools are needed in the world and societies where children are born into today. The yoga interventions should ideally be evidence-based when possible; to be accompanied by empirical research and user participation. Research projects need to be multi-disciplinary and preferably consist of both quantitative and qualitative research methodologies in order to develop this field of yoga research.

Thus, we present the following recommendations: (1) we propose that pre-schools, schools, and community centers offer yoga as part of the opportunities for children and young people to enjoy learning and practicing it from an early age. Obviously, the results of such an investment can be observed over the long-term by having future generations experience less stress. (2) We recommend both pre- and post-intervention studies to ascertain the impact of yoga programs. (3) It is also important to develop a theoretical framework relating children and young people’s mental health and well-being to adequate self-regulation processes [cf. Ref. (46, 47)], in order to create a better theoretical understanding of the potential effects of yoga. (4) Finally, we find it essential to develop policies initiating yoga in schools and training teachers to practice yoga with children.

In terms of the next steps, we suggest that future yoga studies identify gaps in research themes, such as curriculum development, training modules, yoga, and therapeutic correlational and developmental studies. Hopefully, then yoga research and interventions become an attractive choice for research councils and policy planners as an investment in socio-economic and human resource development toward a better society.

## Conflict of Interest Statement

The authors declare that the research was conducted in the absence of any commercial or financial relationships that could be construed as a potential conflict of interest.

## Footnotes

^1^See http://ec.europa.eu/youth/policy/eu_youth_report_en.htm

^2^Such media technologies include computers, laptops, digital television, cellular or smart phones, game consoles PlayStation, Wii, Xbox), and mobile game consoles (Nintendo, PSP, MP3 player, iPad, etc.).

^3^When children are mentioned in this article, the term often refers to children and young people or those under 18 years old. We are of course aware that media use habits vary enormously according to age.
